# Childhood and Adolescent Obesity: A Review

**DOI:** 10.3389/fped.2020.581461

**Published:** 2021-01-12

**Authors:** Alvina R. Kansra, Sinduja Lakkunarajah, M. Susan Jay

**Affiliations:** ^1^Division of Endocrinology, Diabetes and Metabolism, Department of Pediatrics, Medical College of Wisconsin, Milwaukee, WI, United States; ^2^Division of Adolescent Medicine, Department of Pediatrics, Medical College of Wisconsin Affiliated Hospitals, Milwaukee, WI, United States; ^3^Division of Adolescent Medicine, Department of Pediatrics, Medical College of Wisconsin, Milwaukee, WI, United States

**Keywords:** obesity, childhood, review (article), behavior, adolescent

## Abstract

Obesity is a complex condition that interweaves biological, developmental, environmental, behavioral, and genetic factors; it is a significant public health problem. The most common cause of obesity throughout childhood and adolescence is an inequity in energy balance; that is, excess caloric intake without appropriate caloric expenditure. Adiposity rebound (AR) in early childhood is a risk factor for obesity in adolescence and adulthood. The increasing prevalence of childhood and adolescent obesity is associated with a rise in comorbidities previously identified in the adult population, such as Type 2 Diabetes Mellitus, Hypertension, Non-alcoholic Fatty Liver disease (NAFLD), Obstructive Sleep Apnea (OSA), and Dyslipidemia. Due to the lack of a single treatment option to address obesity, clinicians have generally relied on counseling dietary changes and exercise. Due to psychosocial issues that may accompany adolescence regarding body habitus, this approach can have negative results. Teens can develop unhealthy eating habits that result in Bulimia Nervosa (BN), Binge- Eating Disorder (BED), or Night eating syndrome (NES). Others can develop Anorexia Nervosa (AN) as they attempt to restrict their diet and overshoot their goal of “being healthy.” To date, lifestyle interventions have shown only modest effects on weight loss. Emerging findings from basic science as well as interventional drug trials utilizing GLP-1 agonists have demonstrated success in effective weight loss in obese adults, adolescents, and pediatric patients. However, there is limited data on the efficacy and safety of other weight-loss medications in children and adolescents. Nearly 6% of adolescents in the United States are severely obese and bariatric surgery as a treatment consideration will be discussed. In summary, this paper will overview the pathophysiology, clinical, and psychological implications, and treatment options available for obese pediatric and adolescent patients.

## Introduction

Obesity is a complex issue that affects children across all age groups (1–3). One-third of children and adolescents in the United States are classified as either overweight or obese. There is no single element causing this epidemic, but obesity is due to complex interactions between biological, developmental, behavioral, genetic, and environmental factors (4). The role of epigenetics and the gut microbiome, as well as intrauterine and intergenerational effects, have recently emerged as contributing factors to the obesity epidemic (5, 6). Other factors including small for gestational age (SGA) status at birth, formula rather than breast feeding in infancy, and early introduction of protein in infant's dietary intake have been reportedly associated with weight gain that can persist later in life (6–8). The rising prevalence of childhood obesity poses a significant public health challenge by increasing the burden of chronic non-communicable diseases (1, 9).

Obesity increases the risk of developing early puberty in children (10), menstrual irregularities in adolescent girls (1, 11), sleep disorders such as obstructive sleep apnea (OSA) (1, 12), cardiovascular risk factors that include Prediabetes, Type 2 Diabetes, High Cholesterol levels, Hypertension, NAFLD, and Metabolic syndrome (1, 2). Additionally, obese children and adolescents can suffer from psychological issues such as depression, anxiety, poor self-esteem, body image and peer relationships, and eating disorders (13, 14).

So far, interventions for overweight/obesity prevention have mainly focused on behavioral changes in an individual such as increasing daily physical exercise or improving quality of diet with restricting excess calorie intake (1, 15, 16). However, these efforts have had limited results. In addition to behavioral and dietary recommendations, changes in the community-based environment such as promotion of healthy food choices by taxing unhealthy foods (17), improving lunch food quality and increasing daily physical activity at school and childcare centers, are extra measures that are needed (16). These interventions may include a ban on unhealthy food advertisements aimed at children as well as access to playgrounds and green spaces where families can feel their children can safely recreate. Also, this will limit screen time for adolescents as well as younger children.

However, even with the above changes, pharmacotherapy and/or bariatric surgery will likely remain a necessary option for those youth with morbid obesity (1). This review summarizes our current understanding of the factors associated with obesity, the physiological and psychological effects of obesity on children and adolescents, and intervention strategies that may prevent future concomitant issues.

## Definition of Childhood Obesity

Body mass index (BMI) is an inexpensive method to assess body fat and is derived from a formula derived from height and weight in children over 2 years of age (1, 18, 19). Although more sophisticated methods exist that can determine body fat directly, they are costly and not readily available. These methods include measuring skinfold thickness with a caliper, Bioelectrical impedance, Hydro densitometry, Dual-energy X-ray Absorptiometry (DEXA), and Air Displacement Plethysmography (2).

BMI provides a reasonable estimate of body fat indirectly in the healthy pediatric population and studies have shown that BMI correlates with body fat and future health risks (18). Unlike in adults, Z-scores or percentiles are used to represent BMI in children and vary with the age and sex of the child. BMI Z-score cut off points of >1.0, >2.0, and >3.0 are recommended by the World Health Organization (WHO) to define at risk of overweight, overweight and obesity, respectively (19). However, in terms of percentiles, overweight is applied when BMI is >85th percentile <95th percentile, whereas obesity is BMI > 95th percentile (20–22). Although BMI Z-scores can be converted to BMI percentiles, the percentiles need to be rounded and can misclassify some normal-weight children in the under or overweight category (19). Therefore, to prevent these inaccuracies and for easier understanding, it is recommended that the BMI Z-scores in children should be used in research whereas BMI percentiles are best used in the clinical settings (20).

As BMI does not directly measure body fat, it is an excellent screening method, but should not be used solely for diagnostic purposes (23). Using 85th percentile as a cut off point for healthy weight may miss an opportunity to obtain crucial information on diet, physical activity, and family history. Once this information is obtained, it may allow the provider an opportunity to offer appropriate anticipatory guidance to the families.

## Pathophysiology of Obesity

**Figure 1:**
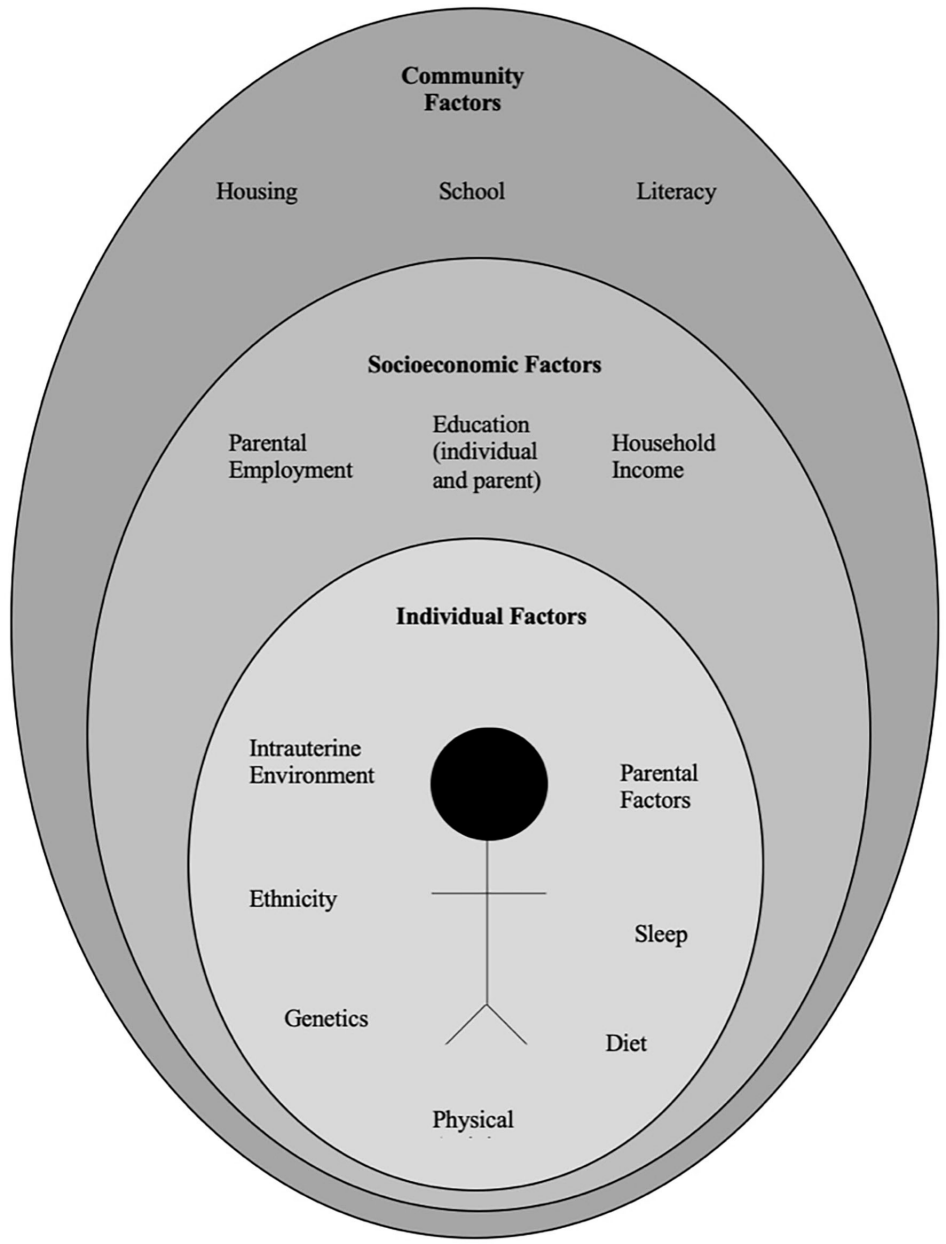
Multidimensional factors contributing to child and adolescent obesity.

The pathophysiology of obesity is complex that results from a combination of individual and societal factors. At the individual level, biological, and physiological factors in the presence of ones' own genetic risk influence eating behaviors and tendency to gain weight (1). Societal factors include influence of the family, community and socio-economic resources that further shape these behaviors (Figure 1) (3, 24).

### Biological Factors

**Figure 2:**
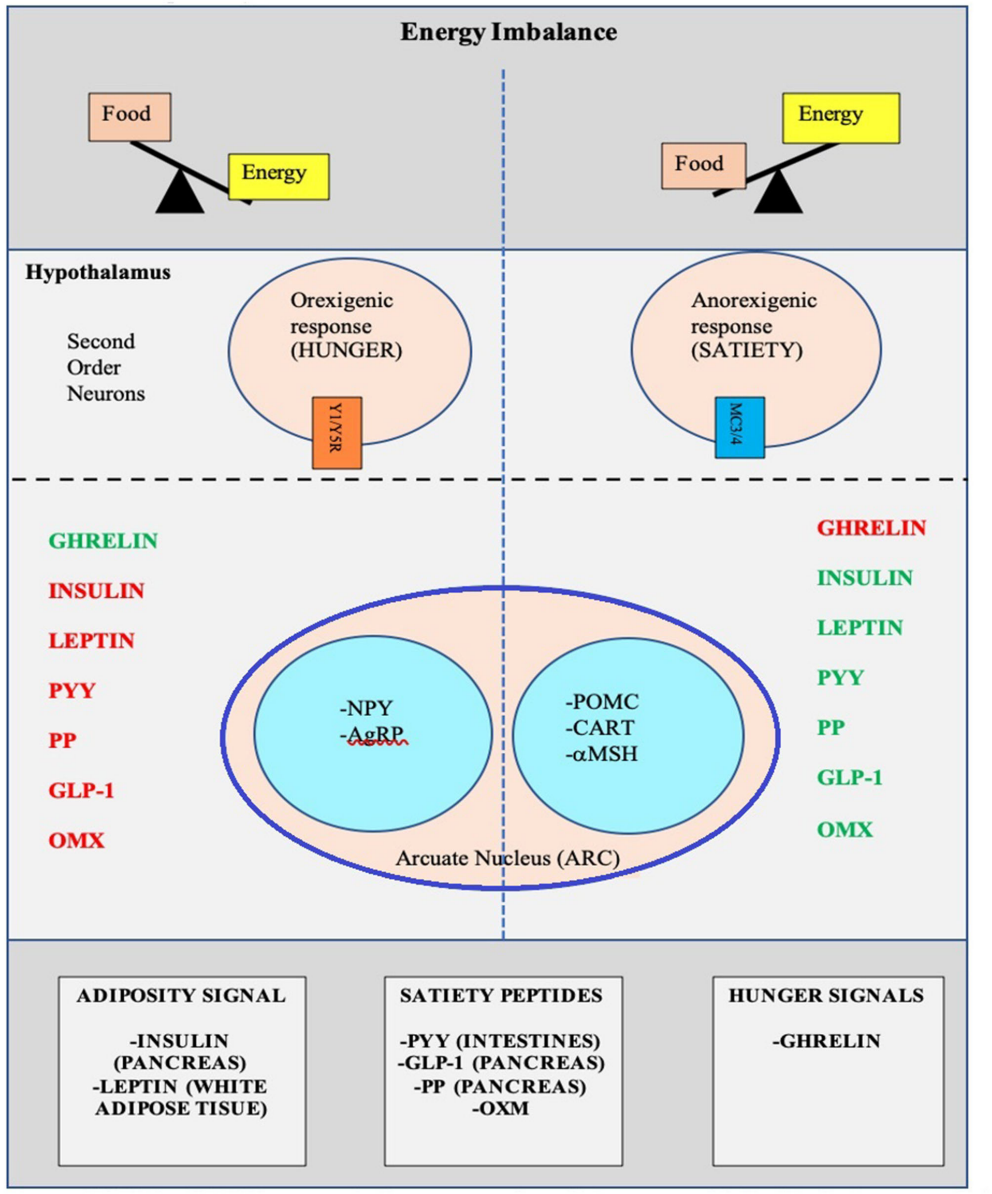
Pictorial representation of the Hunger-Satiety pathway^a^ and the various hormones^b^ involved in the pathway. a, Y1/Y5R and MC3/4 are second order neuro receptors which are responsible in either the hunger or satiety pathway. Neurons in the ARC include: NPY, Neuropeptide Y; AgRP, Agouti-Related Peptide; POMC, Pro-Opiomelanocortin; CART, Cocaine-and Amphetamine-regulated Transcript; α-MSH, α-Melanocyte Stimulating Hormone. b, PYY, Peptide YY; PP, Pancreatic Polypeptide; GLP-1, Glucagon-Like Peptide- I; OMX, Oxyntomodulin.

There is a complex architecture of neural and hormonal regulatory control, the Gut-Brain axis, which plays a significant role in hunger and satiety (Figure 2). Sensory stimulation (smell, sight, and taste), gastrointestinal signals (peptides, neural signals), and circulating hormones further contribute to food intake (25–27).

The hypothalamus is the crucial region in the brain that regulates appetite and is controlled by key hormones. Ghrelin, a hunger-stimulating (orexigenic) hormone, is mainly released from the stomach. On the other hand, leptin is primarily secreted from adipose tissue and serves as a signal for the brain regarding the body's energy stores and functions as an appetite -suppressing (anorexigenic) hormone. Several other appetite-suppressing (anorexigenic) hormones are released from the pancreas and gut in response to food intake and reach the hypothalamus through the brain-blood barrier (BBB) (28–32). These anorexigenic and orexigenic hormones regulate energy balance by stimulating hunger and satiety by expression of various signaling pathways in the arcuate nucleus (ARC) of the hypothalamus (Figure 2) (28, 33). Dysregulation of appetite due to blunted suppression or loss of caloric sensing signals can result in obesity and its morbidities (34).

Emotional dysfunction due to psychiatric disorders can cause stress and an abnormal sleep-wake cycles. These modifications in biological rhythms can result in increased appetite, mainly due to ghrelin, and can contribute to emotional eating (35).

Recently, the role of changes in the gut microbiome with increased weight gain through several pathways has been described in literature (36, 37). The human gut serves as a host to trillions of microorganisms, referred to as gut microbiota. The dominant gut microbial phyla are Firmicutes, Bacteroidetes, Actinobacteria, Proteobacteria, Fusobacteria, and Verrucomicrobia, with Firmicutes and Bacteroidetes representing 90% of human gut microbiota (5, 38). The microbes in the gut have a symbiotic relationship within their human host and provide a nutrient-rich environment. Gut microbiota can be affected by various factors that include gestational age at birth, mode of infant delivery, type of neonatal and infant feeding, introduction of solid food, feeding practices and external factors like antibiotic use (5, 38). Also, the maturation of the bacterial phyla that occurs from birth to adulthood (39), is influenced by genetics, environment, diet, lifestyle, and gut physiology and stabilizes in adulthood (5, 39, 40). Gut microbiota is unique to each individual and plays a specific role in maintaining structural integrity, and the mucosal barrier of the gut, nutrient metabolism, immune response, and protection against pathogens (5, 37, 38). In addition, the microbiota ferments the indigestible food and synthesizes other essential micronutrients as well as short chain fatty acids (SCFAs') (40, 41). Dysbiosis or imbalance of the gut microbiota, in particularly the role of SCFA has been linked with the patho-physiology of obesity (36, 38, 41, 42). SCFAs' are produced by anaerobic fermentation of dietary fiber and indigestible starch and play a role in mammalian energy metabolism by influencing gut-brain communication axis. Emerging evidence has shown that increased ratio of Firmicutes to Bacteroidetes causes increased energy extraction of calories from diets and is evidenced by increased production of short chain fatty acids (SCFAs') (43–45). However, this relationship is not affirmed yet, as a negative relationship between SCFA levels and obesity has also been reported (46). Due to the conflicting data, additional randomized control trials are needed to clarify the role of SCFA's in obese and non-obese individuals.

The gut microbiota also has a bidirectional interaction with the liver, and various additional factors such as diet, genetics, and the environment play a key role in this relationship. The Gut- Liver Axis is interconnected at various levels that include the mucus barrier, epithelial barrier, and gut microbiome and are essential to maintain normal homeostasis (47). Increased intestinal mucosal permeability can disrupt the gut-liver axis, which releases various inflammatory markers, activates an innate immune response in the liver, and results in a spectrum of liver diseases that include hepatic steatosis, non-alcoholic steatohepatitis (NASH), cirrhosis, and hepatocellular carcinoma (HCC) (48, 49).

Other medical conditions, including type 2 Diabetes Mellitus, Metabolic Syndrome, eating disorders as well as psychological conditions such as anxiety and depression are associated with the gut microbiome (50–53).

### Genetic Factors

Genetic causes of obesity can either be monogenic or polygenic types. Monogenic obesity is rare, mainly due to mutations in genes within the leptin/melanocortin pathway in the hypothalamus that is essential for the regulation of food intake/satiety, body weight, and energy metabolism (54). Leptin regulates eating behaviors, the onset of puberty, and T-cell immunity (55). About 3% of obese children have mutations in the leptin (*LEP*) gene and the leptin receptor (LEPR) and can also present with delayed puberty and immune dysfunction (55, 56). Obesity caused by other genetic mutations in the leptin-melanocortin pathway include proopiomelanocortin (POMC) and melanocortin receptor 4 (MC4R), brain-derived neurotrophic factor (BDNF), and the tyrosine kinase receptor B (NTRK2) genes (57, 58). Patients with monogenic forms generally present during early childhood (by 2 years old) with severe obesity and abnormal feeding behaviors (59). Other genetic causes of severe obesity are Prader Willi Syndrome (PWS), Alström syndrome, Bardet Biedl syndrome. Patients with these syndromes present with additional characteristics, including cognitive impairment, dysmorphic features, and organ-specific developmental abnormalities (60). Individuals who present with obesity, developmental delay, dysmorphic features, and organ dysfunction should receive a genetics referral for further evaluation.

Polygenic obesity is the more common form of obesity, caused by the combined effect of multiple genetic variants. It is the result of the interplay between genetic susceptibility and the environment, also known as the Gene-Environment Interaction (GEI) (61–64). Genome-wide association studies (GWAS) have identified gene variants [single nucleotide polymorphism (SNPs)] for body mass index (BMI) that likely act synergistically to affect body weight (65). Studies have identified genetic variants in several genes that may contribute to excessive weight gain by increasing hunger and food intake (66–68). When the genotype of an individual confers risk for obesity, exposure to an obesogenic environment may promote a state of energy imbalance due to behaviors that contribute to conserving rather than expending energy (69, 70). Research studies have shown that obese individuals have a genetic variation that can influence their actions, such as increased food intake, lack of physical activity, a decreased metabolism, as well as an increased tendency to store body fat (63, 66, 67, 69, 70).

Recently the role of epigenetic factors in the development of obesity has emerged (71). The epigenetic phenomenon may alter gene expression without changing the underlying DNA sequence. In effect, epigenetic changes may result in the addition of chemical tags known as methyl groups, to the individual's chromosomes. This alteration can result in a phenomenon where critical genes are primed to on and off regulate. Complex physiological and psychological adjustment occur during infancy and can thereafter set the stage for health vs. disease. Developmental origins of health and disease (DOHaD) shows that early life environment can impact the risk of chronic diseases later in life due to fetal programming secondary to epigenetic changes (72). Maternal nutrition during the prenatal or early postnatal period may trigger these epigenetic changes and increase the risk for chronic conditions such as obesity, metabolic and cardiovascular disease due to epigenetic modifications that may persist and cause intergenerational effect on the health children and adults (58, 73, 74). Similarly, adverse childhood experiences (ACE) have been linked to a broad range of negative outcomes through epigenetic mechanisms (75) and promote unhealthy eating behaviors (76, 77). Other factors such as diet, physical activity, environmental and psychosocial stressors can cause epigenetic changes and place an individual at risk for weight gain (78).

### Developmental Factors

Eating behaviors evolve over the first few years of life. Young children learn to eat through their direct experience with food and observing others eating around them (79). During infancy, feeding defines the relationship of security and trust between a child and the parent. Early childhood eating behaviors shift to more self-directed control due to rapid physical, cognitive, communicative, and social development (80). Parents or caregivers determine the type of food that is made available to the infant and young child. However, due to economic limitations and parents having decreased time to prepare nutritious meals, consumption of processed and cheaper energy-dense foods have occurred in Western countries. Additionally, feeding practices often include providing large or super-sized portions of palatable foods and encouraging children to finish the complete meal (clean their plate even if they do not choose to), as seen across many cultures (81, 82). Also, a segment of parents are overly concerned with dietary intake and may pressurize their child to eat what they perceive as a healthy diet, which can lead to unintended consequences (83). Parents' excessive restriction of food choices may result in poor self-regulation of energy intake by their child or adolescent. This action may inadvertently promote overconsumption of highly palatable restricted foods when available to the child or adolescent outside of parental control with resultant excessive weight gain (84, 85).

During middle childhood, children start achieving greater independence, experience broader social networks, and expand their ability to develop more control over their food choices. Changes that occur in the setting of a new environment such as daycare or school allow exposure to different food options, limited physical activity, and often increased sedentary behaviors associated with school schedules (24). As the transition to adolescence occurs, physical and psychosocial development significantly affect food choices and eating patterns (25). During the teenage years, more independence and interaction with peers can impact the selection of fast foods that are calorically dense. Moreover, during the adolescent years, more sedentary behaviors such as video and computer use can limit physical exercise. Adolescence is also a period in development with an enhanced focus on appearance, body weight, and other psychological concerns (86, 87).

### Environmental Factors

Environmental changes within the past few decades, particularly easy access to high-calorie fast foods, increased consumption of sugary beverages, and sedentary lifestyles, are linked with rising obesity (88). The easy availability of high caloric fast foods, and super-sized portions, are increasingly common choices as individuals prefer these highly palatable and often less expensive foods over fruits and vegetables (89). The quality of lunches and snacks served in schools and childcare centers has been an area of debate and concern. Children and adolescents consume one-third to one-half of meals in the above settings. Despite policies in place at schools, encouraging foods, beverages, and snacks that are deemed healthier options, the effectiveness of these policies in improving children's dietary habits or change in obesity rate has not yet been seen (90). This is likely due to the fact that such policies primarily focus on improving dietary quality but not quantity which can impact the overweight or obese youth (91). Policies to implement taxes on sugary beverages are in effect in a few states in the US (92) as sugar and sugary beverages are associated with increased weight gain (2, 3). This has resulted in reduction in sales of sugary drinks in these states, but the sales of these types of drinks has risen in neighboring states that did not implement the tax (93). Due to advancements in technology, children are spending increased time on electronic devices, limiting exercise options. Technology advancement is also disrupting the sleep-wake cycle, causing poor sleeping habits, and altered eating patterns (94). A study published on Canadian children showed that the access to and night-time use of electronic devices causes decreased sleep duration, resulting in excess body weight, inferior diet quality, and lower physical activity levels (95).

Infant nutrition has gained significant popularity in relation to causing overweight/obesity and other diseases later in life. Breast feeding is frequently discussed as providing protection against developing overweight/obesity in children (8). Considerable heterogeneity has been observed in studies and conducting randomized clinical trials between breast feeding vs. formula feeding is not feasible (8). Children fed with a low protein formula like breast milk are shown to have normal weight gain in early childhood as compared to those that are fed formulas with a high protein load (96). A recent Canadian childbirth cohort study showed that breast feeding within first year of life was inversely associated with weight gain and increased BMI (97). The effect was stronger if the child was exclusively breast fed directly vs. expressed breast milk or addition of formula or solid food (97). Also, due to the concern of poor growth in preterm or SGA infants, additional calories are often given for nutritional support in the form of macronutrient supplements. Most of these infants demonstrate “catch up growth.” In fact, there have been reports that in some children the extra nutritional support can increase the risk for overweight/obesity later in life. The association, however, is inconsistent. Recently a systemic review done on randomized controlled trials comparing the studies done in preterm and SGA infants with feeds with and without macronutrient supplements showed that macronutrient supplements may increase weight and length in toddlers but did not show a significant increase in the BMI during childhood (98). Increased growth velocity due to early introduction of formula milk and protein in infants' diet, may influence the obesity pathways, and can impact fetal programming for metabolic disease later in life (99).

General pediatricians caring for children with overweight/obesity, generally recommend endocrine testing as parents often believe that there may be an underlying cause for this condition and urge their primary providers to check for conditions such as thyroid abnormalities. Endocrine etiologies for obesity are rarely identified and patients with underlying endocrine disorders causing excessive weight gain usually are accompanied by attenuated growth patterns, such that a patient continues to gain weight with a decline in linear height (100). Various endocrine etiologies that one could consider in a patient with excessive weight gain in the setting of slow linear growth: severe hypothyroidism, growth hormone deficiency, and Cushing's disease/syndrome (58, 100).

## Clinical-Physiology of Pediatric Obesity

**Figure 3:**
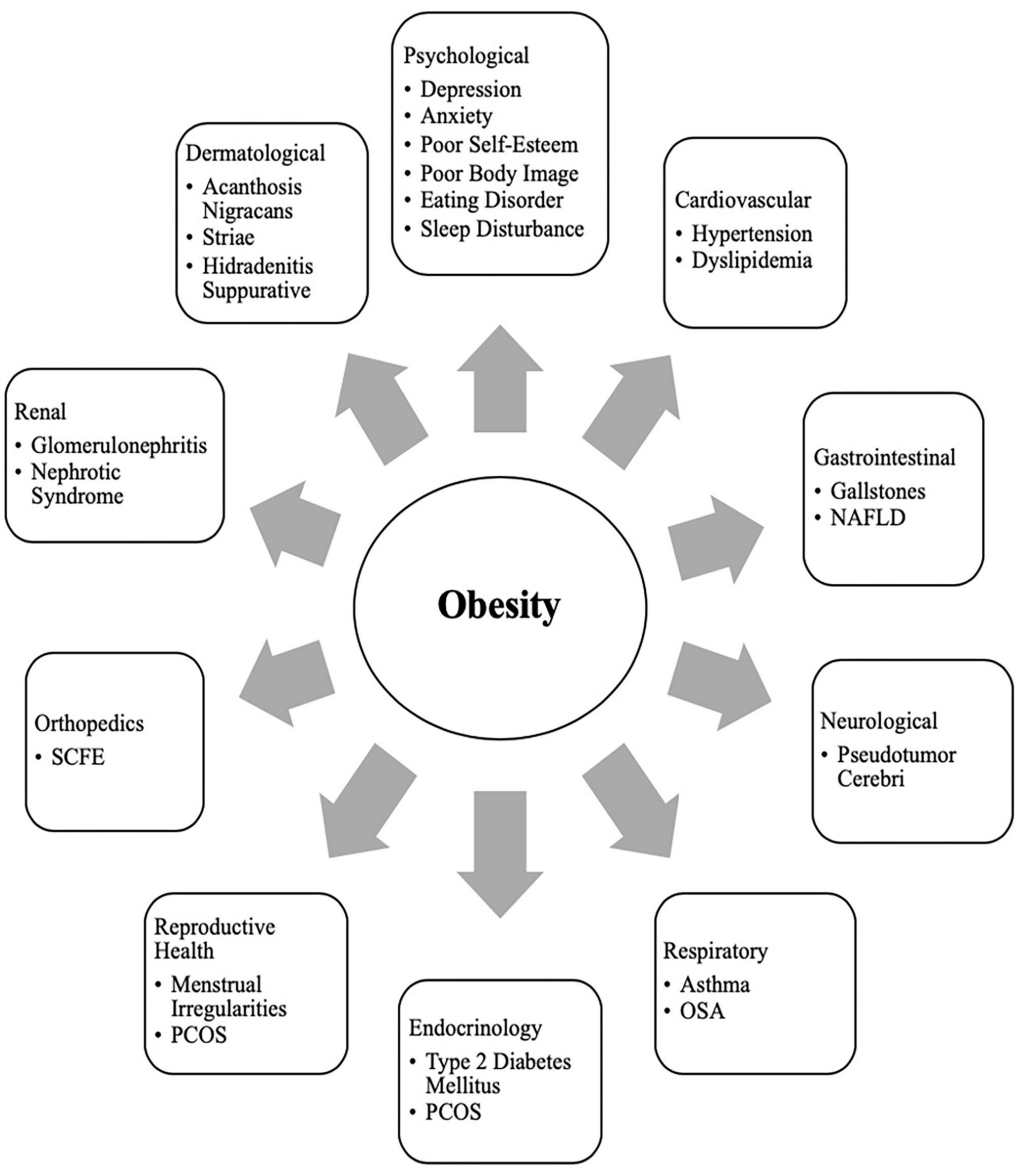
Obesity related co-morbidities^a^ in children and adolescents. a, NAFLD, Non-Alcoholic Fatty Liver Disease; SCFE, Slipped Capital Femoral Epiphysis; PCOS, Polycystic Ovary Syndrome; OSA, Obstructive Sleep Apnea.

It is a well-known fact that early AR(increased BMI) before the age of 5 years is a risk factor for adult obesity, obesity-related comorbidities, and metabolic syndrome (101–103). Typically, body mass index (BMI) declines to a minimum in children before it starts increasing again into adulthood, also known as AR. Usually, AR happens between 5 and 7 years of age, but if it occurs before the age of 5 years is considered early AR. Early AR is a marker for higher risk for obesity-related comorbidities. These obesity-related health comorbidities include cardiovascular risk factors (hypertension, dyslipidemia, prediabetes, and type 2 diabetes), hormonal issues, orthopedic problems, sleep apnea, asthma, and fatty liver disease (Figure 3) (9).

## Clinical Comorbidities of Obesity in Children

### Growth and Puberty

Excess weight gain in children can influence growth and pubertal development (10). Childhood obesity can cause prepubertal acceleration of linear growth velocity and advanced bone age in boys and girls (104). Hyperinsulinemia is a normal physiological state during puberty, but children with obesity can have abnormally high insulin levels (105). Leptin resistance also occurs in obese individuals who have higher leptin levels produced by their adipose tissue (55, 106). The insulin and leptin levels can act on receptors that impact the growth plates with a resultant bone age advancement (55).

Adequate nutrition is essential for the typical timing and tempo of pubertal onset. Excessive weight gain can initiate early puberty, due to altered hormonal parameters (10). Obese children may present with premature adrenarche, thelarche, or precocious puberty (PP) (107). The association of early pubertal changes with obesity is consistent in girls, and is well-reported; however, data is sparse in boys (108). One US study conducted in racially diverse boys showed obese boys had delayed puberty, whereas overweight boys had early puberty as compared to normal-weight boys (109). Obese girls with PP have high leptin levels (110, 111). Healthy Lifestyle in Europe by Nutrition in Adolescence (HELENA) is a cross-sectional study and suggested an indirect relationship between elevated leptin levels, early puberty, and cardiometabolic and inflammatory markers in obese girls (112). Additionally, obese girls with premature adrenarche carry a higher risk for developing polycystic ovary syndrome (PCOS) in the future (113, 114).

### Sleep Disorders

Obesity is an independent risk factor for obstructive sleep apnea (OSA) in children and adolescents (12, 115). Children with OSA have less deleterious consequences in terms of cardiovascular stress of metabolic syndrome when compared to adolescents and adults (116, 117). In children, abnormal behaviors and neurocognitive dysfunction are the most critical and frequent end-organ morbidities associated with OSA (12). However, in adolescents, obesity and OSA can independently cause oxidative systemic stress and inflammation (118, 119), and when this occurs concurrently, it can result in more severe metabolic dysfunction and cardiovascular outcomes later in life (120).

### Other Comorbidities

Obesity is related to a clinical spectrum of liver abnormalities such as NAFLD (121); the most important cause of liver disease in children (122–124). NAFLD includes steatosis (increased liver fat without inflammation) and NASH (increased liver fat with inflammation and hepatic injury). While in some adults NAFLD can progress to an end-stage liver disease requiring liver transplant (125, 126), the risk of progression during childhood is less well-defined (127). NAFLD is closely associated with metabolic syndrome including central obesity, insulin resistance, type 2 diabetes, dyslipidemia, and hypertension (128).

Obese children are also at risk for slipped capital femoral epiphysis (SCFE) (129), and sedentary lifestyle behaviors may have a negative influence on the brain structure and executive functioning, although the direction of causality is not clear (130, 131).

## Clinical Comorbidities of Obesity in Adolescents

### Menstrual Irregularities and PCOS

At the onset of puberty, physiologically, sex steroids can cause appropriate weight gain and body composition changes that should not affect normal menstruation (132, 133). However, excessive weight gain in adolescent girls can result in irregular menstrual cycles and puts them at risk for PCOS due to increased androgen levels. Additionally, they can have excessive body hair (hirsutism), polycystic ovaries, and can suffer from distorted body images (134, 135). Adolescent girls with PCOS also have an inherent risk for insulin resistance irrespective of their weight. However, weight gain further exacerbates their existing state of insulin resistance and increases the risk for obesity-related comorbidities such as metabolic syndrome, and type 2 diabetes. Although the diagnosis of PCOS can be challenging at this age due to an overlap with predictable pubertal changes, early intervention (appropriate weight loss and use of hormonal methods) can help restore menstrual cyclicity and future concerns related to childbearing (11).

### Metabolic Syndrome and Sleep Disorders

Metabolic syndrome (MS) is a group of cardiovascular risk factors characterized by acanthosis nigricans, prediabetes, hypertension, dyslipidemia, and non-alcoholic steatohepatitis (NASH), that occurs from insulin resistance caused by obesity (136). Diagnosis of MS in adults requires at least three out of the five risk factors: increased central adiposity, hypertension, hyperglycemia, hypertriglyceridemia, or low HDL level. Definitions to diagnose MS are controversial in younger age groups, and many definitions have been proposed (136). This is due to the complex physiology of growth and development during puberty, which causes significant overlap between MS and features of normal growth. However, childhood obesity is associated with an inflammatory state even before puberty (137). In obese children and adolescents, hyperinsulinemia during puberty (138, 139) and unhealthy sleep behaviors increase MS's risk and severity (140). Even though there is no consensus on diagnosis regarding MS in this age group, when dealing with obese children and adolescents, clinicians should screen them for MS risk factors and sleep behaviors and provide recommendations for weight management.

### Social Psychology of Pediatric Obesity in Children and Adolescents

Obese children and adolescents may experience psychosocial sequelae, including depression, bullying, social isolation, diminished self-esteem, behavioral problems, dissatisfaction with body image, and reduced quality of life (13, 141). Compared with normal-weight counterparts, overweight/obesity is one of the most common reasons children and adolescents are bullied at school (142). The consequence of stigma, bullying, and teasing related to childhood obesity are pervasive and can have severe implications for emotional and physical health and performance that can persist later in life (13).

**Figure 4:**
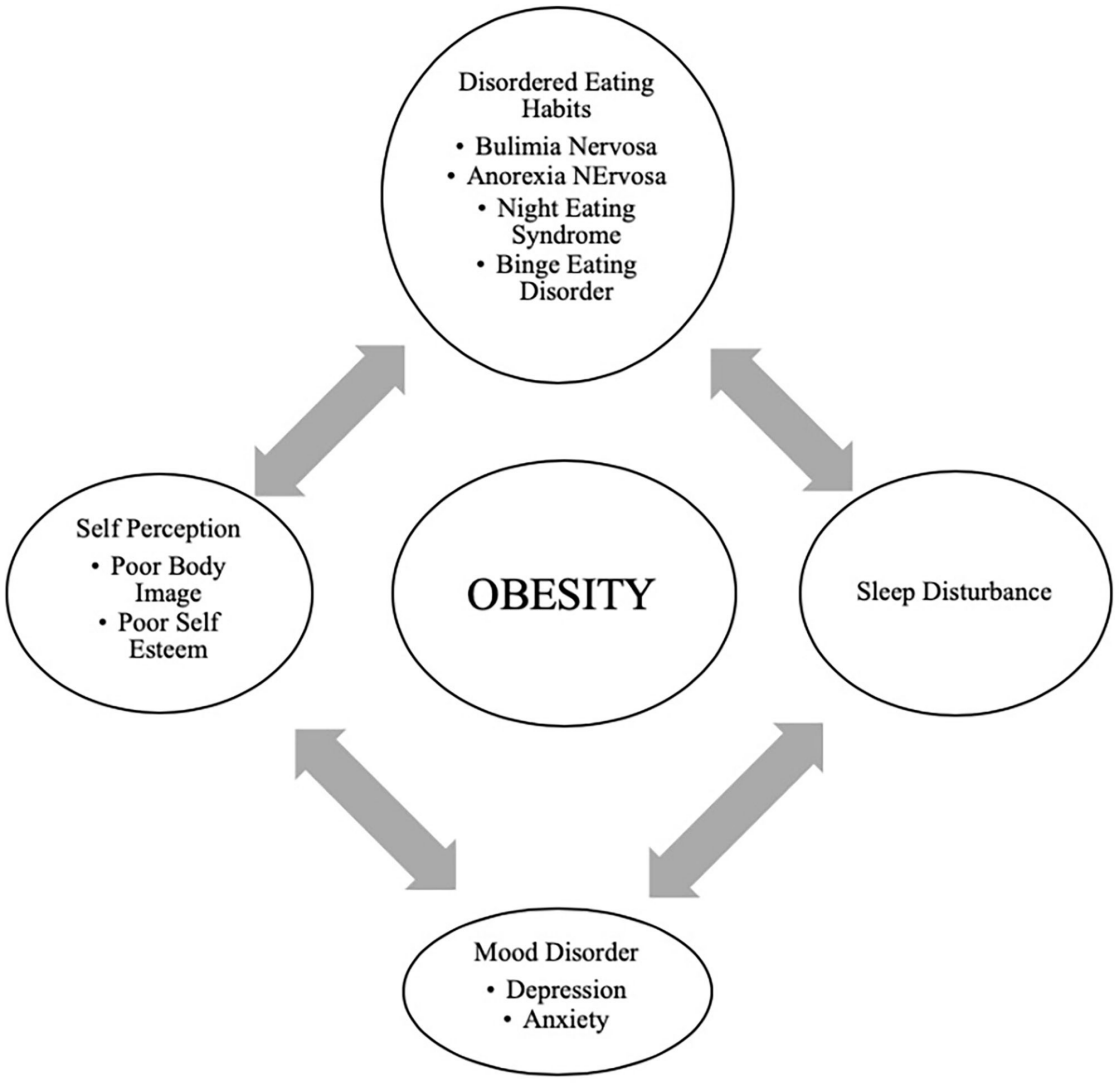
Bidirectional relationship of different psychological outcomes of obesity.

In adolescents, psychological outcomes associated with obesity are multifactorial and have a bidirectional relationship (Figure 4). Obese adolescents due to their physique may have a higher likelihood of psychosocial health issues, including depression, body image/dissatisfaction, lower self-esteem, peer victimization/bullying, and interpersonal relationship difficulties. They may also demonstrate reduced resilience to challenging situations compared to their non-obese/overweight counterparts (9, 143–146). Body image dissatisfaction has been associated with further weight gain but can also be related to the development of a mental health disorder or an eating disorder (ED) or disorder eating habits (DEH). Mental health disorders such as depression are associated with poor eating habits, a sedentary lifestyle, and altered sleep patterns. ED or DEH that include anorexia nervosa (AN), bulimia nervosa (BN), binge-eating disorder (BED) or night eating syndrome (NES) may be related to an individual's overvaluation of their body shape and weight or can result during the treatment for obesity (147–150). The management of obesity can place a patient at risk of AN if there is a rigid focus on caloric intake or if a patient overcorrects and initiates obsessive self-directed dieting. Healthcare providers who primarily care for obese patients, usually give the advice to diet to lose weight and then maintain it. However, strict dieting (hypocaloric diet), which some patients may later engage in can lead to an eating disorder such as anorexia nervosa (151). This behavior leads to a poor relationship with food, and therefore, adolescents perseverate on their weight and numbers (152).

Providers may not recognize DEHs when a morbidly obese patient loses the same weight as a healthy weight individual (149). It may appear as a positive result with families and others praising the individual without realizing that this youth may be engaging in destructive behaviors related to weight control. Therefore, it is essential to screen regarding the process of how weight loss was achieved (144, 150).

Support and attention to underlying psychological concerns can positively affect treatment, overall well-being, and reduce the risk of adult obesity (150). The diagram above represents the complexity of the different psychological issues which can impact the clinical care of the obese adolescent.

Eating family meals together can improve overall dietary intake due to enhanced food choices mirrored by parents. It has also may serve as a support to individuals with DEHs if there is less attention to weight and a greater focus on appropriate, sustainable eating habits (148).

## Treatment

### Prevention and Anticipatory Guidance

It is essential to recognize and provide preventive measures for obesity during early childhood and adolescence (100, 153, 154). It is well-established that early AR is a risk factor for adult obesity (66–68). Therefore, health care providers caring for the pediatric population need to focus on measures such as BMI but provide anticipatory guidance regarding nutritional counseling without stigmatizing or judging parents for their children's overweight/obesity (155). Although health care providers continue to pursue effective strategies to address the obesity epidemic; ironically, they frequently exhibit weight bias and stigmatizing behaviors. Research has demonstrated that the language that health care providers use when discussing a patient's body weight can reinforce stigma, reduce motivation for weight loss, and potentially cause avoidance of routine preventive care (155). In adolescents, rather than motivating positive changes, stigmatizing language regarding weight may negatively impact a teen and result in binge eating, decreased physical activity, social isolation, avoidance of health care services, and increased weight gain (156, 157). Effective provider-patient communication using motivational interviewing techniques are useful to encourage positive behavior changes (155, 158).

Anticipatory guidance includes educating the families on healthy eating habits and identifying unhealthy eating practices, encouraging increased activity, limiting sedentary activities such as screen time. Lifestyle behaviors in children and adolescents are influenced by many sectors of our society, including the family (Figure 1) (3, 24). Therefore, rather than treating obesity in isolation as an individual problem, it is crucial to approach this problem by focusing on the family unit. Family-based multi-component weight loss behavioral treatment is the gold standard for treating childhood obesity, and it is having been found useful in those between 2 and 6 years old (150, 159). Additionally, empowering the parents to play an equal role in developing and implementing an intervention for weight management has shown promising results in improving the rate of obesity by decreasing screen time, promoting healthy eating, and increasing support for children's physical activity (160, 161).

When dietary/lifestyle modifications have failed, the next option is a structured weight -management program with a multidisciplinary approach (15). The best outcomes are associated with an interdisciplinary team comprising a physician, dietician, and psychologist generally 1–2 times a week (15, 162). However, this treatment approach is not effective in patients with severe obesity (122). Although healthier lifestyle recommendations for weight loss are the current cornerstone for obesity management, they often fail. As clinicians can attest, these behavioral and dietary changes are hard to achieve, and all too often is not effective in patients with severe obesity. Failure to maintain substantial weight loss over the long term is due to poor adherence to the prescribed lifestyle changes as well as physiological responses that resist weight loss (163). American TV hosts a reality show called “The Biggest Loser” that centers on overweight and obese contestants attempting to lose weight for a cash prize. Contestants from “The Biggest Loser” competition, had metabolic adaptation (MA) after drastic weight loss, regained more than they lost weight after 6 years due to a significant slow resting metabolic rate (164). MA is a physiological response which is a reduced basal metabolic rate seen in individuals who are losing or have lost weight. In MA, the body alters how efficient it is at turning the food eaten into energy; it is a natural defense mechanism against starvation and is a response to caloric restriction. Plasma leptin levels decrease substantially during caloric restriction, suggesting a role of this hormone in the drop of energy expenditure (165).

### Pharmacological Management

The role of pharmacological therapy in the treatment of obesity in children and adolescents is limited.

Orlistat is the only FDA approved medication for weight loss in 12-18-year-olds but has unpleasant side effects (166). Another medicine, Metformin, has been used in children with signs of insulin resistance, may have some impact on weight, but is not FDA approved (167). The combination of phentermine/topiramate (Qsymia) has been FDA approved for weight loss in obese individuals 18 years and older. In studies, there has been about 9–10% weight loss over 2 years. However, caution must be taken in females as it can lead to congenital disabilities, especially with use in the first trimester of pregnancy (167).

GLP-1 agonists have demonstrated great success in effective weight loss and are approved by the FDA for adult obesity (168–170). A randomized control clinical trial recently published showed a significant weight loss in those using liraglutide (3.0 mg)/day plus lifestyle therapy group compared to placebo plus lifestyle therapy in children between the ages of 12–18 years (171).

Recently during the EASL conference, academic researchers and industry partners presented novel interventions targeting different gut- liver axis levels that include intestinal content, intestinal microbiome, intestinal mucosa, and peritoneal cavity (47). The focus for these therapeutic interventions within the gut-liver axis was broad and ranged anywhere from newer drugs protecting the intestinal mucus lining, restoring the intestinal barriers and improvement in the gut microbiome. One of the treatment options was Hydrogel technology which was shown to be effective toward weight loss in patients with metabolic syndrome. Hydrogel technology include fibers and high viscosity polysaccharides that absorb water in the stomach and increasing the volume, thereby improving satiety (47). Also, a clinical trial done in obese pregnant mothers using Docosahexaenoic acid (DHA) showed that the mothers' who got DHA had children with lower adiposity at 2 and 4 years of age (172). Recently the role of probiotics in combating obesity has emerged. Probiotics are shown to alter the gut microbiome that improves intestinal digestive and absorptive functions of the nutrients. Intervention including probiotics may be a possible solution to manage pediatric obesity (173, 174). Additionally, the role of Vitamin E for treating the comorbidities of obesity such as diabetes, hyperlipidemia, NASH, and cardiovascular risk, has been recently described (175, 176). Vitamin E is a lipid- soluble compound and contains both tocopherols and tocotrienols. Tocopherols have lipid-soluble antioxidants properties that interact with cellular lipids and protects them from oxidation damage (177). In metabolic disease, certain crucial pathways are influenced by Vitamin E and some studies have summarized the role of Vitamin E regarding the treatment of obesity, metabolic, and cardiovascular disease (178). Hence, adequate supplementation of Vitamin E as an appropriate strategy to help in the treatment of the prevention of obesity and its associated comorbidities has been suggested. Nonetheless, some clinical trials have shown contradictory results with Vitamin E supplementation (177). Although Vitamin E has been recognized as an antioxidant that protects from oxidative damage, however, a full understanding of its mechanism of action is still lacking.

### Bariatric Surgery

Bariatric surgery has gained popularity since the early 2000s in the management of severe obesity. If performed earlier, there are better outcomes for reducing weight and resolving obesity-related comorbidities in adults (179–182). Currently, the indication for bariatric in adolescents; those who have a BMI >35 with at least one severe comorbidity (Type 2 Diabetes, severe OSA, pseudotumor cerebri or severe steatohepatitis); or BMI of 40 or more with other comorbidities (hypertension, hyperlipidemia, mild OSA, insulin resistance or glucose intolerance or impaired quality of life due to weight). Before considering bariatric surgery, these patients must have completed most of their linear growth and participated in a structured weight-loss program for 6 months (159, 181, 183). The American Society for Metabolic and Bariatric Surgery (AMBS) outlines the multidisciplinary approach that must be taken before a patient undergoing bariatric surgery. In addition to a qualified bariatric surgeon, the patient must have a pediatrician or provider specialized in adolescent medicine, endocrinology, gastroenterology and nutrition, registered dietician, mental health provider, and exercise specialist (181). A mental health provider is essential as those with depression due to obesity or vice versa may have persistent mental health needs even after weight loss surgery (184).

Roux-en-Y Gastric Bypass (RYGB), laparoscopic Sleeve Gastrectomy (LSG), and Gastric Banding are the options available. RYGB and LSG currently approved for children under 18 years of age (166, 181, 185). At present, gastric banding is not an FDA recommended procedure in the US for those under 18y/o. One study showed some improvements in BMI and severity of comorbidities but had multiple repeat surgeries and did not believe a suitable option for obese adolescents (186).

Compared to LSG, RYGB has better outcomes for excess weight loss and resolution of obesity-related comorbidities as shown in studies and clinical trials (183, 184, 187). Overall, LSG is a safer choice and may be advocated for more often (179–181). The effect on the Gut-Brain axis after Bariatric surgery is still inconclusive, especially in adolescents, as the number of procedures performed is lower than in adults. Those who underwent RYGB had increased fasting and post-prandial PYY and GLP-1, which could have contributed to the rapid weight loss (185); this effect was seen less often in patients with gastric banding (185). Another study in adult patients showed higher bile acid (BA) subtype levels and suggested a possible BA's role in the surgical weight loss response after LSG (188). Adolescents have lower surgical complication rates than their adult counterparts, hence considering bariatric surgery earlier rather than waiting until adulthood has been entertained (180). Complications after surgery include nutritional imbalance in iron, calcium, Vitamin D, and B12 and should be monitored closely (180, 181, 185). Although 5-year data for gastric bypass in very obese teens is promising, lifetime outcome is still unknown, and the psychosocial factors associated with adolescent adherence post-surgery are also challenging and uncertain.

## Conclusion

Obesity in childhood and adolescence is not amenable to a single easily modified factor. Biological, cultural, and environmental factors such as readily available high-density food choices impact youth eating behaviors. Media devices and associated screen time make physical activity a less optimal choice for children and adolescents. This review serves as a reminder that the time for action is now. The need for interventions to change the obesogenic environment by instituting policies around the food industry and in the schools needs to be clarified. In clinical trials GLP-1 agonists are shown to be effective in weight loss in children but are not yet FDA approved. Discovery of therapies to modify the gut microbiota as treatment for overweigh/obesity through use of probiotics or fecal transplantation would be revolutionary. For the present, ongoing clinical research efforts in concert with pharmacotherapeutic and multidisciplinary lifestyle programs hold promise.

## Author Contributions

AK, SL, and MJ contributed to the conception and design of the study. All authors contributed to the manuscript revision, read, and approved the submitted version.

## Conflict of Interest

The authors declare that the research was conducted in the absence of any commercial or financial relationships that could be construed as a potential conflict of interest.
